# Circulating miR-148b-3p and miR-27a-3p can be potential biomarkers for diagnosis of pre-diabetes and type 2 diabetes: integrating experimental and in-silico approaches

**DOI:** 10.1186/s12902-022-01120-5

**Published:** 2022-08-17

**Authors:** Elnaz Ghoreishi, Seyedeh Zahra Shahrokhi, Faranak Kazerouni, Ali Rahimipour

**Affiliations:** 1 Department of Medical Laboratory Sciences, School of Allied Medical Sciences, Shahid Beheshti University of Medical Sciences, Tehran, Iran; 2 Department of Biochemistry, School of Medicine, AJA University of Medical Sciences, Tehran, Iran; 3 Department of Clinical Biochemistry, School of Medicine, Shahid Beheshti University of Medical Sciences, Tehran, Iran

**Keywords:** miRNAs, Type 2 diabetes, miR-148b-3p, miR-27a-3p, Diagnostic value

## Abstract

**Background:**

In view of the growing global prevalence of type 2 diabetes (T2D), detection of prediabetes and type 2 diabetes in the early stages is necessary to reduce the risk of developing diabetes, prevent the progression of the disease, and dysfunction of different organs. Since miRNAs are involved in the initiation and progression of numerous pathogenic processes, including diabetes, in the present study, we aimed to investigate the expression of miR-148b-3p and miR-27a-3p in prediabetic and T2D patients and to evaluate the diagnostic potential of these miRNAs.

**Methods:**

We evaluated the expression of miR-148b-3p and miR-27a-3p in the plasma of three groups: 20 prediabetic patients, 20 T2D patients, and 20 healthy controls. The biochemical parameters were determined by the auto-analyzer. The possible target genes of these miRNAs were identified using an in-silico approach.

**Results:**

Our results showed that, as compared to the healthy controls, there was a significant up regulation and down regulation in the expression of miR-148b-3p and miR-27a-3p in the T2D patients, respectively. The results of receiver operating characteristic curve analysis also suggested that miR-148b-3p acted successfully in discriminating the prediabetic and diabetic patients from the control group. According to *in-silico* analysis, miRs influence biological pathways involved in T2DM development, such as insulin signaling.

**Conclusions:**

The miR148b-3p and miR-27a-3p expression levels were deregulated in diabetes and pre-diabetes. Furthermore, miR-148b-3p showed significant ability in discriminating between diabetic and healthy individuals, suggesting a potential diagnostic use of miR-148b-3p in the detection of T2D.

## Background

The high prevalence and multitude of devastating side effects along with the multifactorial characteristics put major obstacles in the way of successful management of diabetes, a disease identified as a global health concern [1, 2]. When it comes to type 2 diabetes, early detection plays a fundamental role in disease control. The sooner the disease is detected at its earlier stages, the fewer the devastating effects that increased blood glucose would impose on other organs [3]. Since the first clinical description of diabetes, an ever-increasing number of factors have been identified as being involved in the disease pathogenesis. However, attempts to find a precise molecule that would be responsible for diabetes development are still ongoing. In recent years, advances in genome-wide studies have dramatically revolutionized the molecular landscape of diabetes research by identifying the association between single nucleotide polymorphisms (SNPs) in genes encoding proteins and transcription factors and the development of type 2 diabetes [1]. Since the majority of these SNPs have been found in non-coding regions, it could be postulated that non-coding RNAs, primarily microRNA (miRNA) may be the answer to the mystery of diabetes pathogenesis.

miRNAs are a new class of small (19 to 25 nucleotides) single-stranded RNAs that bind to the complementary regions in target mRNAs and robustly stop the process of translation by their degradation [4]. A mounting body of evidence suggests that miRNAs affect the regulation of a wide range of intracellular biological processes such as cell differentiation, apoptosis, necrosis, proliferation and migration [5]. Moreover, the involvement of these non-coding RNAs in the pathogenesis of several chronic and inflammatory diseases such as human cancers and autoimmune disease has been well-identified [6, 7]. Apart from the regulatory roles of miRNAs, their high stability as well as their wide presence in body fluids has attracted tremendous attention and highlighted their importance as valid biomarkers [8]. Another benefit of miRNAs is that their alteration could be detected prior to the complete progression of disease, introducing them as a potential biomarker for early detection [9]. Furthermore, since miRNAs can be easily suppressed by therapeutic interventions, they can be used as an option for alternative subsequent treatments [10, 11]. For the nonce, the alteration in the expression level of a multitude of miRNAs has been used as a diagnostic marker in different types of diseases such as various cancers [12, 13], hepatitis B [8] and last but not least, diabetes [14]. Although the results from laboratory experiments have provided significant evidence for the prominent diagnostic application of miRNAs in type 2 diabetes mellitus (T2DM), there is still a long path ahead. To the best of our knowledge, the present study evaluated alterations in expression of miR-148b-3p and miR-27a-3p in type 2 diabetic and pre-diabetic patients in comparison to healthy controls in Iranian individuals for the first time.

## Methods

### Sample collection

In this pilot study, 60 participants were selected from individuals who were referred to Shohada Tajrish Hospital (20 type 2 diabetic patients, 20 pre-diabetics and 20 healthy subjects). Participants were 35 years or older. Diabetic patients were designated according to the World Health Organization and were described with fasting blood glucose (FBG) > 126 mg/ dl and glycated hemoglobin (HbA1c) > 6.5%. Pre-diabetes was diagnosed with FBG: 100–126 mg/dl and HbA1c: 5.7–6.5%. The healthy control group consisted of individuals with FBG < 100 mg/dl and HbA1c < 5.7%. The exclusion criteria for the patients included the presence of inflammatory diseases, malignancies, chronic diseases, renal and hepatic diseases, endocrine disorders and history of tobacco and alcohol consumption. Written informed consent was obtained from all patients and controls.

The demographic characteristics of the patients including age, sex, consumption of anti-diabetic medications, body mass index (BMI), blood pressure, and the duration of diabetes were registered by an interviewer‐administered questionnaire. After an overnight (12 h) fast, 10 cc of blood was collected from the subjects and used for plasma and serum separation.

### Biochemical parameter measurements

After 12 h fasting, biochemical parameters including fasting blood glucose (FBG), high-density lipoprotein cholesterol (HDL-c), total cholesterol (TC), low-density lipoprotein cholesterol (LDL-c), triglycerides (TG), creatinine (Cr) and blood urea nitrogen (BUN) of samples were measured by an auto-analyzer (Hitachi, Tokyo, Japan). FBS was measured by the enzymatic glucose oxidase method of the Man company (Iran, Cat. No: 613003). TC is measured enzymatically using the cholesterol reagent of the Man company (Iran, Cat. No: 613007). TG are analyzed enzymatically with cholesterol using reagents from the same manufacturer (Iran, Cat. No: 613018). Direct HDL cholesterol reagent was obtained from the Man company (Iran, Direct HDL, Cat. No: 613066). Serum LDL-c was estimated using William T Friedewald formula based on fasting plasma measurements of TC, HDL-c and TG ([LDL] = [TC]—[HDL]—[TG]/5). Serum creatinine was measured by the Jaffe method of the Man company (Iran, cat.no. 613027). Serum BUN was measured by the Urease/GLDH UV- Kinetic method of the Man company (Iran, cat.no. 613020). HbA1c levels were measured by HPLC (Waters, Milford, MA, USA). BMI was calculated as weight in kilograms divided by the square of the person’s height in meters (kg/m2).

### mRNA extraction from plasma and cDNA synthesis

Whole blood samples (5 ml) were collected in the tubes containing EDTA from participants who had fasted for 12 h. Within 1 h of blood collection, samples were centrifuged at 1900 g for 10 min at 4 ºC, and plasma was carefully transferred into microcentrifuge tubes. A second centrifugation at 1600 g for 10 min at 4 °C was performed to remove any cellular debris and to reduce contamination of cell free nucleic acids (gDNA and RNA) derived from damaged blood cells. Plasma was stored at -80 °C until further analysis. MiRNAs were extracted from 250 μl of plasma using the MiRNeasy Mini kit (Cat. No: 217184, Qiagen, Germany). Extracted RNA was eluted with 14 μl of RNase-free water according to the manufacturer’s recommendation. We determined the yield and purity of the miRNAs based on 260/280 and 260/230 ratios using Nanodrop ND-1000 spectrophotometry (Nanodrop Technologies, Wilmington, Delaware, USA). For cDNA synthesis, the reverse transcription (RT) reaction was performed using the PrimeScript RT Reagent Kit (Cat. No: RR037A, Takara Bio, Japan). The mixture was composed of 4 μl 5X PCR buffer, 1 μl RT Enzyme, 1 μl random hexamers, 1 μg RNA per reaction and RNase free-water up to 20 μl per reaction. This mixture was heated for 15 min at 37 °C, and 5 s at 85 °C. The final cDNA was stored at -20 °C, until the evaluation of gene expression.

### Real-time PCR analysis

To evaluate the alteration in the expression of the genes, synthesized cDNA was subjected to a Rotor-Gene real-time thermal cycler (Qiagen, Valencia, CA) using SYBR Premix Ex Taq Reagent (Cat. No: RR820L, Takara Bio, Japan). A 20 µL reaction, which possessed 10 μl of SYBR Green master mix, 2 μl of cDNA, 0.5 μl of each forward and reverse primer (10 pmol) and 7 μl of nuclease-free water was amplified in a thermal cycler. For each target gene, primer efficiency was estimated from the standard curve using four consecutives 1:10 dilutions of the cDNA sample. U6 snoRNA was amplified as a housekeeping gene. The real-time PCR program included an initial activation step for 30 s at 5 °C followed by 40 cycles, including a denaturation step for 5 s at 95 °C and a combined annealing/extension step for 20 s at 58 °C. Melting curves were analyzed to verify the single PCR product of each primer. All the individual samples were run in duplicates. The primers used in this study are listed in Table 1.

**Table 1:** Primer sequences for qRT-PCR analysis

	**Stem loop RT primer**	**Forward Primer**
**MiR-27a-3p**	GTCGTATCCAGTGCAGGGTCCGAGGTATTCGCACTGGATACGGCGGT	GCTTCACAGTGGCTAAGTT
**MiR-148b-3p**	GTCGTATCCAGTGCAGGGTCCGAGGTATTCGCACTGGATACGACAAAGT	CGGCTCAGTGCACTACAGA
**U6 snoRNA**	GTCGTATCCAGTGCAGGGTCCGAGGTATTCGCACTGGATACGAAAAAT	AAATTGGAACGATACAGAGAAG

### MicroRNA target prediction

To investigate the role of miR-27a-3p and miR-148b-3p in T2DM progression and to identify their target genes, bioinformatic analyses were used. TargetScan (http://www.targetscan.org), as well as the DIANA-Tools (http://diana.imis.athena-innovation.gr) databases, were used to predict microRNA targets. The interactions between microRNA and target genes were visualized using the Cytoscape software.

### Statistical analysis

Descriptive statistics were expressed using the mean ± standard deviation (SD). The Kruskal–Wallis test was used to determine if there were statistically significant differences between clinical and demographic variables. The correlation of miR-148b-3p and miR-27a-3p with biochemical variables was evaluated by Spearman correlation. The relative expression levels of miR-148b-3p and miR-27a-3p were calculated by using the 2^−∆∆CT^ method among the three patient groups. Receiver operating characteristic (ROC) curve analysis and comparison of the derived area under the curve (AUC) was performed to evaluate the possible roles of miR-148b-3p and miR-27a-3p in the diagnosis of diabetes and pre-diabetes from healthy controls. The Wilson-Brown model in GraphPad prism software was used for ROC curve analysis. All analyses were performed using GraphPad Prism 8 Statistical Software (GraphPad Software, La Jolla, CA, USA). *P*-values less than 0.05 were considered statistically significant.

## Results

### Basic characteristics of the study subjects

The clinical characteristics of the patients are summarized in Table 2. There were significant differences between the diabetic and pre-diabetic patients in FBG (*P* < 0.001), HbA1c (*P* < 0.001), and TG (*P* < 0.001) compared with the control group. Basic characteristics such as age (*P* = 0.035), BMI (*P* = 0.043), FBG (*P* < 0.001), HbA1c (*P* < 0.001), TG (*P* < 0.001) and Cr (*P* = 0.043) were significantly different among the groups. In terms of sex, height, weight, BP, HDL, LDL and BUN, no significant differences were seen among the groups (*P* > 0.05) (Table 2).

**Table 2:** Baseline characteristics of different patient groups included in the study

Variable	Control	Diabetes patients	Pre-diabetes patients	*P*-value
Sex				
Female	7 (35.0%)	13 (65.0%)	9 (45.0%)	0.197
Male	13 (65.0%)	7 (35.0%)	11 (55.0%)	
Age groups (years)				
≤ 45	39.33 ± 2.12	40.6 ± 3.78	40.83 ± 3.6	0.68
46–60	50.5 ± 4.59	51.88 ± 4.40	53.85 ± 5.33	
> 61	63.8 ± 3.03	66.16 ± 2.63	65 ± 2.51	
BMI groups (kg/m^2^)				
≤ 25	23.36 ± 1.48	23.68 ± 1.25	23.29 ± 1.44	0.19
25–30	26.59 ± 1.24	27.32 ± 1.17	27.82 ± 1.21	
> 30	32.19 ± 1.40	33.68 ± 2.76	35.2 ± 4.41	
BP (mm Hg)	12.80 ± 1.60	12.58 ± 1.66	11.74 ± 1.52	0.070
FBG (mg/dl)	89.95 ± 7.76	166.05 ± 68.26b	104.35 ± 8.73^b^	< 0.001***
HbA1c (%)	5.43 ± 0.36	8.01 ± 1.64^a^	5.87 ± 0.41^b^	< 0.001***
TG (mg/dl)	132.60 ± 94.79	230.85 ± 100.54^a^	168.85 ± 72.25^b^	< 0.001***
TC (mg/dl)	197.55 ± 38.62	197.85 ± 51.06	193.85 ± 31.67	0.987
HDL (mg/dl)	47.09 ± 14.37	45.71 ± 12.95	46.07 ± 9.41	0.782
LDL (mg/dl)	124.20 ± 33.82	103.61 ± 44.55	116.90 ± 26.17	0.261
Cr (mg/dl)	1.01 ± 0.10	1.13 ± 1.07	1.03 ± 0.18	**0.043**
BUN (mg/dl)	13.95 ± 2.42	15.20 ± 3.82	15.40 ± 3.60	0.486

Numeric data are expressed as Mean ± SD and were compared by the Kruskal–Wallis test and Chi-square test. Categorical data were summarized as frequency (percentage) and were compared by chi-square test

*BMI* Body mass index, *BP* Blood pressure, *HbA1c* glycated hemoglobin, *TG* Triglyceride, *TC* Total cholesterol, *HDL-C* High-density lipoprotein cholesterol, *LDL* Low-density lipoprotein cholesterol, *Cr* Creatinine, *BUN* Blood urea nitrogen.

^a^ *P* < 0.05 Control vs. Diabetes patients

^b^ *P* < 0.05 Control vs. Pre-diabetes patients

### Comparison of expression levels of miR-148b-3p and miR-27a-3p in plasma of healthy, prediabetic and diabetic subjects

**Fig. 1:**
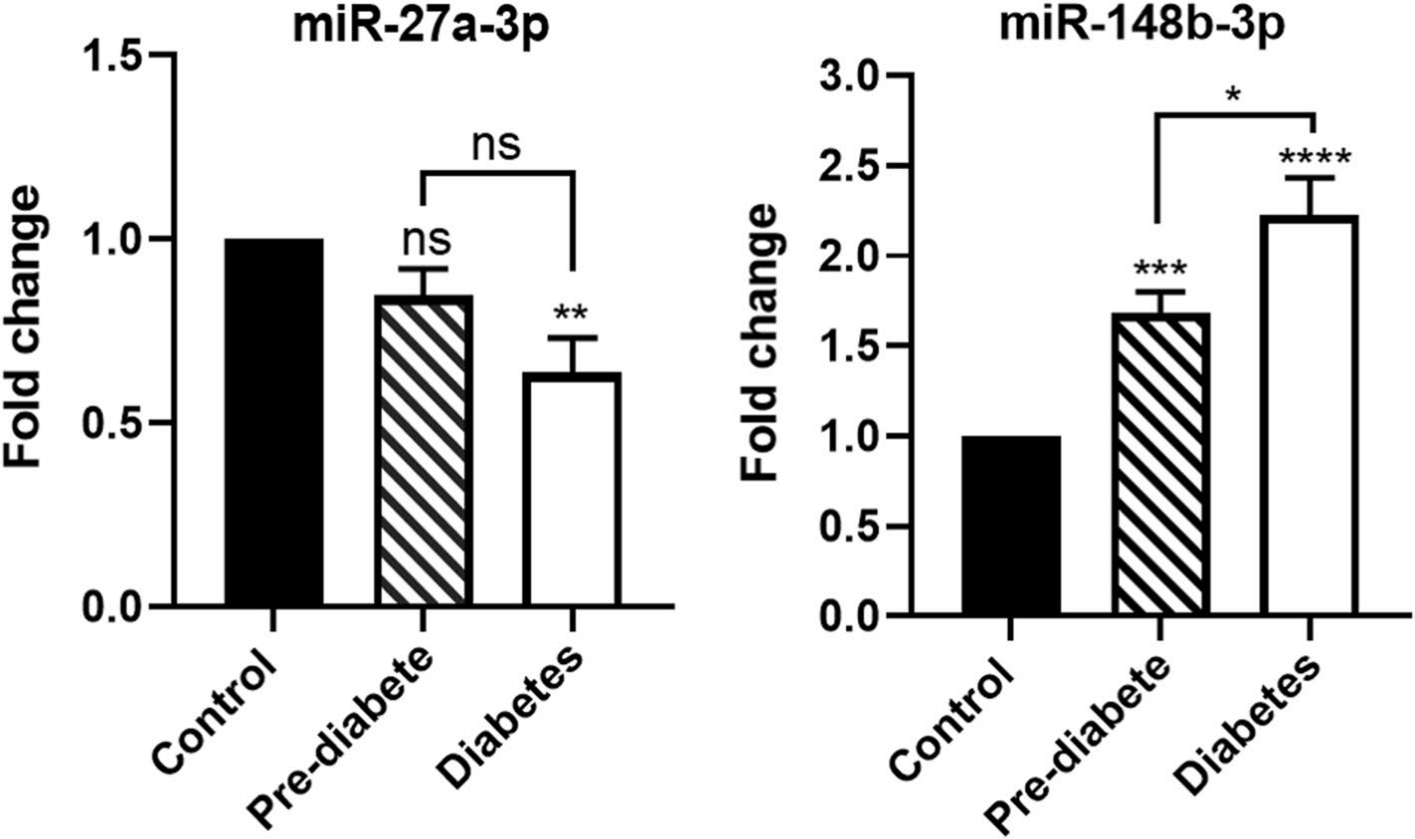
Comparison of miR-27a-3p and miR-148b-3p expressions in the control, pre-diabetic, and diabetic groups. U6 snoRNA used as an internal control

Plasma expression levels of miR-148b-3p and miR-27a-3p in the healthy, prediabetic, and diabetic individuals were detected by Real-Time-PCR. Based on our findings, the relative expression of miR-148b-3p was significantly higher in the diabetic and pre-diabetic patients than in the control group (*P* < 0.0001 and *P* < 0.001, respectively). Furthermore, a significant difference was observed in miR-148b-3p expression between diabetic and pre-diabetic subjects (*P* < 0.01) (Fig. 1). On the other hand, the relative expression of miR-27a-3p was lower in both the diabetic and pre-diabetic groups compared to the control group. Although the expression level of miR-27a-3p in the diabetic group was also lower than that in the pre-diabetic group, this difference was not statistically significant (Fig. 1). The alteration in the expression of miR-27a-3p was only significant between the diabetic and the control group (*P* < 0.01).

### Correlation between miR-148b-3p and miR-27a-3p with anthropometrical factors and clinical variables

The correlation between miR-148b-3p and miR-27a-3p expression with anthropometric factors and clinical variables was evaluated using the Spearman correlation coefficient. As presented in Table 3, miR-148b-3p expression positively correlates with FBG in the prediabetic group (*r* = 0.55, *P* = 0.01). In the T2D group, we also found a positive correlation between the expression of miR-148b-3p and FBG (*r* = 0.37, *P* = 0.004), TC (*r* = 0.55, *P* = 0.01), HDL-c (*r* = 0.52, *P* = 0.01) and LDL-c (*r* = 0.6, *P* = 0.004). Based on the results obtained, miR-27a-3p expression was positively correlated with blood pressure in the control group (*r* = 0.53, *P* = 0.01). In the prediabetic group, there was a negative correlation between the expression of miR-27a-3p and FBG (*r* = -0.45, *P* = 0.04). We failed to find any correlation between the expression of miR-27a-3p and anthropometric factors as well as clinical variables in the diabetic group (Table 3).

**Table 3:** Spearman correlation between biochemical parameters and variables including miRNAs

Variables	miR-27a-3p						miR-148b-3p					
	**Control**		**Pre-diabetes**		**Diabetes**		**Control**		**Pre-diabetes**		**Diabetes**	
	*r*	*P*	*r*	*P*	*r*	*P*	*r*	*P*	*r*	*P*	*r*	*P*
Age	-0.13	0.57	-0.1	0.67	-0.08	0.71	0.01	**0.94**	-0.25	0.28	0.10	0.66
BMI	0.15	0.5	0.17	0.45	0.12	0.58	-0.43	0.05	0.37	0.1	0.05	0.82
BP	0.53	0.01^*^	-0.24	0.3	0.22	0.34	-0.23	0.3	0.16	0.49	0.1	0.65
**Blood Glucose**												
FBG	-0.41	0.07	-0.45	0.04^*^	-0.39	0.08	0.37	0.09	0.55	0.01^*^	0.37	0.004^*^
HbA1c	-0.27	0.23	-0.20	0.38	-0.18	0.43	0.18	0.44	0.01	0.94	0.15	0.08
**Lipid Profile**												
TG	-0.33	0.14	-0.23	0.31	-0.12	0.6	-0.005	0.98	0.4	0.07	0.007	0.9
TC	0.29	0.20	-0.37	0.1	0.36	0.18	-0.09	0.7	0.7	0.53	0.55	0.01^*^
HDL-C	0.27	0.24	0.12	0.6	0.15	0.5	-0.09	0.69	0.11	0.62	0.52	0.01^*^
LDL	0.38	0.09	-0.4	0.07	0.39	0.08	-0.07	0.74	0.22	0.34	0.6	0.004^*^
**Renal Test**												
Cr	-0.49	0.063	0.33	0.173	0.18	0.458	-0.13	0.57	-0.28	0.21	-0.04	0.86
BUN	0.36	0.187	0.40	0.091	0.22	0.346	-0.10	0.65	-0.34	0.13	-0.24	0.30

### Evaluation of the diagnostic values of blood miR-148b-3p and miR-27a-3p

**Fig. 2:**
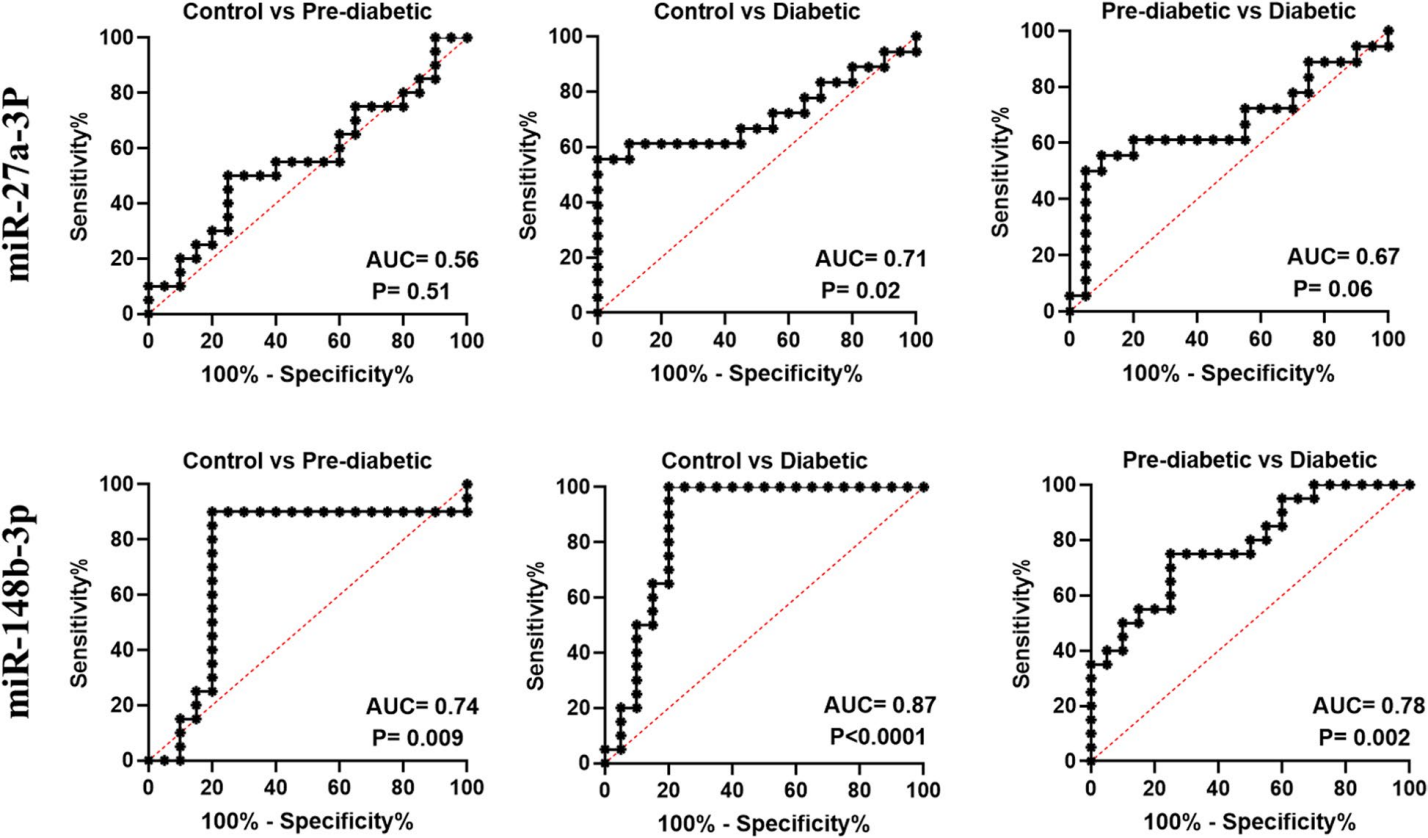
ROC curves analysis of plasma miR-27a-3p and miR-148b-3p for discrimination between the cases of prediabetic, diabetics and the control group. The area under the curve of miR-27a-3p can differentiate the diabetic patients from the control group with an AUC of 0.71 (95% CI 0.53–0.89, *P* = 0.02), while the AUC for discriminating the pre-diabetics from the control group is 0.56 (95% CI 0.37–0.74, *P* = 0.51) and the AUC for discriminating the pre-diabetics from the diabetics is 0.67 (95% CI 0.48–0.85, *P* = 0.06).For miR-148b-3p AUC is of 0.87 (95% CI 0.74–0.99, *P* < 0.0001) for discriminating the T2D patients from the control subjects,for discriminating the pre-diabetic patients from the control group the AUC is 0.74 (95% CI 0.56–0.91, *P* = 0. 009) and the AUC of 0.78 (95% CI 0.64–0.92, *P* = 0.002) can differentiate the pre-diabetic from the diabetic patients

Receiver operating characteristic (ROC) curve analysis was used to evaluate the possible roles of miR-148b-3p and miR-27a-3p in the diagnosis of diabetes and pre-diabetes. As shown in Fig. 2, miR-148b-3p could differentiate the diabetic patients from the control group with an AUC of 0.87 (95% CI 0.74–0.99, *P* < 0.0001) and the pre-diabetic patients from the control group with an AUC of 0.74 (95% CI 0.56–0.91, *P* = 0.009), while the AUC of 0.78 (95% CI 0.64–0.92, *P* = 0.002) could differentiate pre-diabetic from diabetic patients. Regarding miR-27a-3p, as presented in Fig. 2, miR-27a-3p could differentiate the diabetic patients from the control group with an AUC of 0.71 (95% CI 0.53–0.89, *P* = 0.02), while the AUC for discriminating the pre-diabetic patients from the control group was 0.56 (95% CI 0.37–0.74, *P* = 0.51) and the AUC for discriminating pre-diabetic from diabetic individuals was 0.67 (95% CI 0.48–0.85, *P* = 0.06).

### Prediction results

**Fig. 3:**
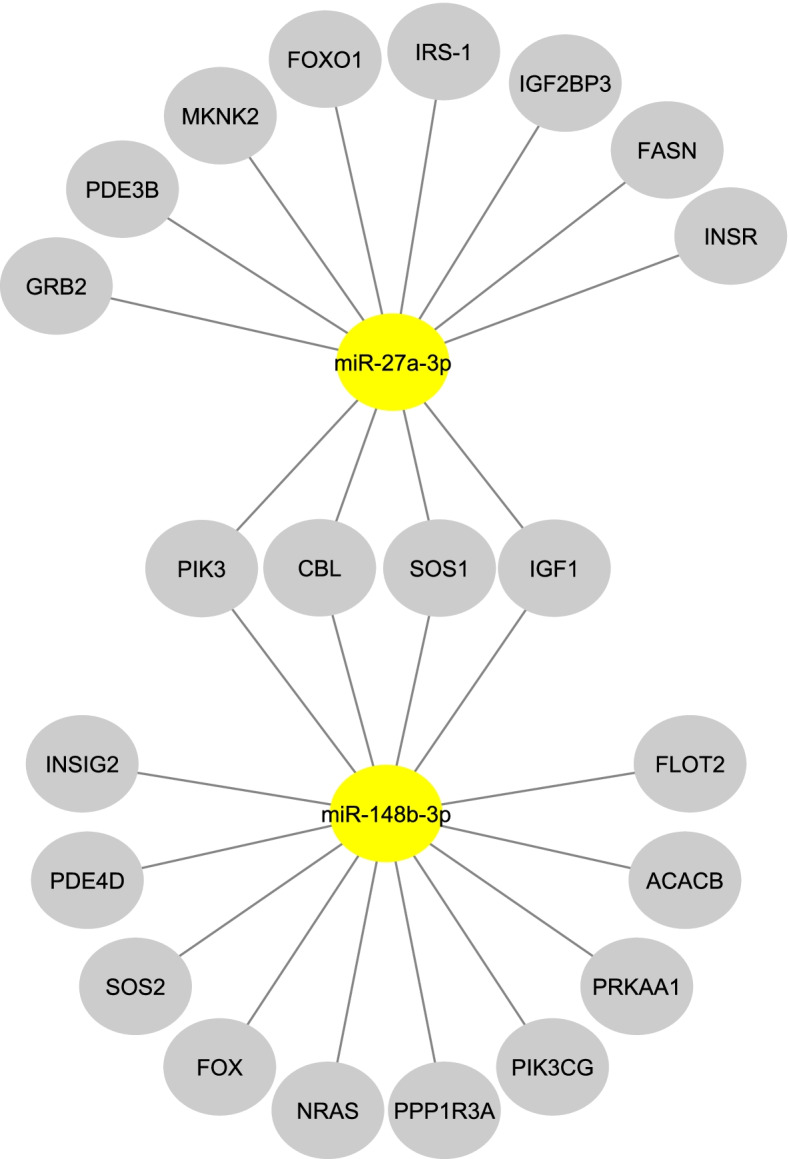
Interactions between miRNAs and target genes. MiRNAs are shown by yellow ellipses, whereas target genes are illustrated by gray ellipses

Our bioinformatic analysis reveals that miR-27a-3p and miR-148b-3p have an effect on biological pathways involved in T2DM pathogenesis, such as insulin signaling pathways (Fig. 3). Out of the total genes, four genes were regulated by two miRNAs. Eight genes were specific to miR-27a-3p and ten genes were specific to miR-148b-3p. These findings support the idea that these miRNAs are targeting genes and pathways involved in diabetes.

## Discussion

Type II Diabetes Mellitus (T2DM) seems to be the most common metabolic disease worldwide, caused either by a defect in insulin secretion from pancreatic β-cells or the lack of responsiveness of insulin-sensitive tissues to the hormone [15]. According to the report released by the International Diabetes Federation (IDF), T2DM is the cause of death of nearly 4.2 million individuals, and the number of victims of this disease is growing day by day. Apart from affecting the quality of life, T2DM is also considered to be a significant risk factor for other diseases such as cardiovascular disease and cancer [16]. Although numerous studies have focused on the mechanisms that participate in the pathogenesis of T2DM, little is known about the disease. Apart from the genetic aberrant, it seems that epigenetic modulations can also be involved in the development of T2DM. Since the first description of microRNAs the footprint of this group of small non-coding RNAs can be traced in different human diseases, and T2DM is no exception. So far, many miRNAs have been identified to be involved in the pathogenesis of T2DM through modulating β-cell differentiation, glucose metabolism, and insulin synthesis [17]. It has also been indicated that, similar to other diseases, miRNAs can also be used as a diagnostic marker for T2DM. In this regard, Shahrokhi *et* al. demonstrated that the miR-145 expression level is deregulated in diabetic and pre-diabetic groups [18]. It was suggested that miR-145-5p displays a significant ability to differentiate diabetics from healthy subjects [18]. In another study, Saeidi *et* al. indicated that the miR-7-5p and miR-33a-5p expression levels were deregulated in diabetes and pre-diabetes and miR-33a-5p showed a significant ability in discriminating between the diabetic and the healthy subjects, suggesting a potential diagnostic use of miRNAs in the detection of type-2 diabetes [19]. Although numerous studies have focused on miRNAs and their association with T2DM, none of these small non-coding RNAs were selected as diagnostic tools, as in some cases, the results were conflicting. The differences in sample size, materials and methodologies, preselected pools of miRNAs as well as the heterogeneous nature of the disease are the main reasons why miRNAs have not reached the clinical settings for T2DM [20]. Given these, in the present study, we aimed to evaluate the diagnostic value of miR-148b-3p and miR-27a-3p in Iranian pre-diabetic and T2DM patients.

In the present study, we found that while the expression of miR-148b-3p was elevated in pre-diabetic and diabetic patients, the expression of miR-27a-3p was significantly down regulated in diabetic patients. Our results also revealed a significant correlation between the expression of these miRNAs and the anthropometric factors of the patients. For the first time, we found a meaningful association between the expression of miR-148b-3p and several biochemical parameters such as FBG, total cholesterol, LDL-c and HDL-c in diabetic patients. In line with our results, Yan *et*.al., found that the serum expression level of miR-148b was significantly higher in the T2D group compared with the controls. The authors suggested that miR-148b may be a potential tool for the early detection of T2DM [21]. MiR-148b-3p is a member of the miRNA-148 family, consisting of miR-148a and miR-148-b, that participate in the regulation of many genes such as SLC2A1, mTORC1 and TNFR2. Chen *et* al. indicated that this family of miRNAs can also regulate methylation of CPG islands [22]. In this regard, miR-148b has been shown to diminish the expression of DNMT1 in β-cells, which is essential for the formation and maturation of the cells [23]. So far, the association between the expression of the miR-148b-3p family and numerous diseases has been investigated. The aberrant over-expression of miR-148a has been reported in autoimmune diseases such as type I diabetes and lupus. In another study, Grieco *et* al. suggested that miR-148a can be secreted from CD14-positive osteoclast precursor cells and negatively regulate the expression of MFB and RNAKL, which in turn increases the differentiation of osteoclasts, an event that leads to bone resorption [24]. Massaro *et* al. conducted massive parallel sequencing and reported that there is a correlation between the expression of miR-148b-3p and the nephropathy complication in diabetic patients [25]. It has also been claimed that miR-148a-3p could regulate both glucose and HbA1c levels in patients [26, 27]. Regulation of immune cells is another mechanism of action of the miR-148 family in the pathogenesis of diabetes [28]. The miR-148 family can also induce insulin resistance by activating immune cells and producing pro-inflammatory cytokines, [29, 30]. Given these and based on the results obtained, it is reasonable to suggest that miR-148b-3p may play a role in the pathogenesis of T2D, at least partly, through accelerating the expression of inflammatory cytokines or by regulating DNA methylation.

MiR-27a-3p is another miRNA that our results showed to be down regulated in diabetic patients, and its expression showed a negative correlation with FBG levels in pre-diabetic patients. MiR-27a-5p is indeed a potent tumor-suppressive miRNA that can regulate the expression of a wide range of genes involved in cell proliferation and migration [31, 32]. MiR-27a may play a critical role in the pathophysiology of obesity-induced insulin resistance in mice by regulating macrophage polarization via inhibiting PPARγ [33]. Among the different downstream targets of miR-27a-5p, PPARγ is one of the most important nuclear receptors that plays a key role in glucose and fatty acid metabolism. Zhang *et* al. have reported the correlation between western diets and the expression of miR-27a-5p. They reported that the up regulated miR-27a-5p induces fatty liver disease by suppressing the expression of PPARγ [34]. Another study also reported that down regulation of miR-27a-5p might be able to abolish hypoxia-induced cardiomyocyte injury through targeting apoptotic and autophagy-related pathways [35]. Chen *et al.*investigated miR-27a expression levels in obese mice fed a high-fat diet (HFD). The authors reported that miR-27a is involved in the PI3K/Akt signaling pathway, resulting in improved glucose uptake and decreased insulin resistance. As a result, miR-27a could be a potential target for the therapy of insulin resistance in obesity and diabetes [36]. MiR-27a-3p can also regulate the expression of several genes, such as RARα, PI3K, FOXO1, MAP2K4, MAPK14, IRS-1, DAGs, TNF-α, IL-10, NOVA1, COX1, COXIV, ROS and ATF3. Of particular interest, since we found that miR-27a-3p is down regulated in pre-diabetic patients, it could be postulated that perhaps the expression of this miRNA is diminished to protect the pancreatic cells from the devastating effects of excessive insulin.

Due to the strong stability of circulating noncoding RNAs in various body fluids such as blood, plasma, serum, saliva, and urine, they can be used as markers for early diagnosis [37]. The ROC curve analysis, which can assess diagnostic precision, was performed to investigate the diagnostic capacity of miR-148b-3p and miR-27a-3p. Based on our results, miR-148b-3p showed an AUC of 0.87 (95% CI 0.74–0.99, *P* < 0.0001) for discriminating the T2D patients from the control subjects. For discriminating the pre-diabetic patients from the control group, the AUC was 0.74 (95% CI 0.56–0.91, *P* = 0. 009) and the AUC of 0.78 (95% CI 0.64–0.92, *P* = 0.002) could differentiate the pre-diabetic from the diabetic patients. Based on the results of the ROC test, we suggest that miR-148b-3p can be used as a marker for early detection of diabetes, especially in individuals who have a family history of T2DM.

It should be noted that in our study, we focused on the alteration in expression of miR-148b-3p and miR-27a-3p in Iranian type 2 diabetes and pre-diabetic patients in comparison to the healthy group. Therefore, to provide a better landscape for the diagnosis of T2DM, the molecular mechanisms recruited by these miRNAs to induce diabetes should be studied more precisely. Furthermore, the limitations of this research included the relatively small sample size and the difference in duration of diabetes. Therefore, to gain a better understanding of the role of miRNAs in T2DM pathogenesis, studies with a larger sample size, diverse ethnicity, and a shorter duration of disease are required.

## Conclusions

Our results demonstrate that miR-148b-3p and miR-27a-3p expression levels are deregulated in diabetes and pre-diabetes. Furthermore, miR-148b-3p showed significant ability in discriminating between diabetic and healthy individuals, suggesting a potential diagnostic use of miR-148b-3p in detection of type-2 diabetes.

## Data Availability

The datasets generated and/or analyzed during the current study are not publicly available but are available from the corresponding author upon reasonable request.
